# A new era in the management of spinal metastasis

**DOI:** 10.3389/fonc.2024.1374915

**Published:** 2024-04-16

**Authors:** Tadatsugu Morimoto, Yu Toda, Michiyuki Hakozaki, Permsak Paholpak, Kazuyuki Watanabe, Kinshi Kato, Masatsugu Tsukamoto, Hirohito Hirata, Yoichi Kaneuchi, Yasunori Tome, Satomi Nagamine, Kotaro Nishida, Hiroo Katsuya, Yoshihiro Matsumoto, Koji Otani, Masaaki Mawatari, Takuya Nikaido

**Affiliations:** ^1^Department of Orthopedic Surgery, Faculty of Medicine, Saga University, Saga, Japan; ^2^Department of Orthopaedic Surgery, Fukushima Medical University School of Medicine, Fukushima, Japan; ^3^Department of Orthopedics, Faculty of Medicine, Khon Kaen University, Khon Kaen, Thailand; ^4^Department of Orthopedic Surgery, Graduate School of Medicine, University of the Ryukyus, Okinawa, Japan; ^5^Division of Hematology, Respiratory Medicine and Oncology, Department of Internal Medicine, Faculty of Medicine, Saga University, Saga, Japan

**Keywords:** spinal metastasis, cancer locomo, multidisciplinary approach, preemptive treatment, minimally invasive spine surgery

## Abstract

Despite the recent advances in cancer treatment, the incidence of patients with spinal metastases continues to grow along with the total number of cancer patients. Spinal metastases can significantly impair activities of daily living (ADL) and quality of life (QOL), compared with other types of bone metastases, as they are characterized with severe pain and paralysis caused by skeletal-related events. Reduced ADL can also lead to treatment limitations as certain anticancer agents and radiation therapy are not compatible treatments; thus, leading to a shorter life expectancy. Consequently, maintaining ADLs in patients with spinal metastases is paramount, and spine surgeons have an integral role to play in this regard. However, neurosurgeon, orthopedic and spinal surgeons in Japan do not have a proactive treatment approach to spinal metastases, which may prevent them from providing appropriate treatment when needed (clinical inertia). To overcome such endemic inertia, it is essential for 1) spine surgeons to understand and be more actively involved with patients with musculoskeletal disorders (cancer locomo) and cancer patients; 2) the adoption of a multidisciplinary approach (coordination and meetings not only with the attending oncologist but also with spine surgeons, radiologists, rehabilitation specialists, and other professionals) to preemptive treatment such as medication, radiotherapy, and surgical treatment; and 3) the integration of the latest findings associated with minimally invasive spinal treatments that have expanded the indications for treatment of spinal metastases and improved treatment outcomes. This heralds a new era in the management of spinal metastases.

## 1 Introduction

In 2016, for the first time, Japan had over 1 million new incidents of cancer per year, which was more than the number of new births (1, 2). This might be explained by Japan’s super-aging society and the advances in cancer treatment (early diagnosis and treatment).

Recent advances in chemotherapy, molecular targeted therapy, immune checkpoint inhibitors, bone-modifying agents (BMA) such as zoledronic acid and denosumab, and radiotherapy have enabled multidisciplinary and comprehensive treatment, extending the lives of patients with cancer worldwide (3). Significant improvements in life expectancy have led to a new era of living with cancer with shortened hospital stays and increased outpatient treatments (4). However, although the prognosis of cancer patients improved, the incidence of metastatic cancer increased as well (5). Spinal metastases are the most frequent bone metastases (50%), and the prevalence of patients with spinal metastases increased in the past years (6). Spinal metastases have been identified in 36% of patients with malignant neoplasms (7). Spinal metastases can significantly reduce activities of daily living (ADL) and quality of life (QOL), compared with other types of bone metastases, as they are accompanied by severe pain and paralysis resulting from skeletal-related events (SREs). Declining ADL can be a significant burden on the life of the patient, leading to a shift in the management focus from curative treatment to life-prolonging palliative care.

The performance status (PS) is extensively utilized in oncology study and practice as a measure of ability to perform daily activities (8). PS 2, “ambulatory and capable of all self-care but unable to carry out any work activities; up and about more than 50% of waking hours,” represents the minimal requirement for chemotherapy to be proposed.

Declining PS can also lead to contraindications for chemotherapy and radiotherapy, resulting in shortened life expectancy. Therefore, the presence of spinal metastases is an important issue for patients, doctors, and society as it has a serious influence on the ADL, QOL, and life expectancy of patients. Furthermore, the population of patients with spinal metastasis is predicted to expand given the super-aged Japanese society and the improvement of imaging technology.

Thus, a paradigm shift in cancer care is currently taking place in Japan, where care is required not only to cure cancer, but also to preserve and improve the QOL of patients with cancer. A shift is being made towards accepting cancer to be a chronically ill condition and aiming to preserve and improve the ADL and QOL of patients with cancer. Consequently, the importance of maintaining ADL and improving the QOL of patients with spinal metastasis has increased, similar to the role of the spine surgeon in patient treatment.

A new era has arrived in which spine surgeons must use their specialist knowledge and skills to contribute as integral team members in cancer care. However, it has been shown that orthopedic surgeons in Japan are not having a proactive approach in the management of patients with bone metastases (1). Clinical inertia is a term that describes this lack of a proactive approach (9). Clinical inertia refers to the failure to start timely treatment and the failure to adapt the treatment in cases where evidence-based treatment targets are not met. In this review, we highlight three methods through which spine surgeons can overcome clinical inertia when treating spinal metastasis patients and become actively involved.

First, spine surgeons must be recognized for the locomotive syndrome in cancer patients (so-called “Cancer Locomo”) (1). The concept of Cancer Locomo, discussed in detail in Section 2, refers to musculoskeletal disorders in cancer patients and contributes to a more active involvement of spine surgeons in the management of spinal metastases. Ultimately, this will improve not only the QOL of patients with locomotor cancer, but also their prognosis.

In Section 3, we discuss the necessity of introducing a multidisciplinary approach. The momentum for the adoption of a multidisciplinary approach involving collaboration among multiple disciplines, such as radiology, orthopedics, oncology, palliative care, and rehabilitation for the prevention and treatment of SRE in patients with spinal metastases has been reported in various studies and clinical guidelines that are gaining momentum (10–16). With respect to spinal metastases, the importance of coordination between spine surgeons, radiologists, and oncologists is critical as failure to do so may delay treatment.

Third, spinal surgeons should consider the advancements in minimally invasive treatments to better manage spinal metastases. Advances in drug treatment, radiotherapy, and minimally invasive spine surgery (MISS) have expanded the management alternatives for patients with spinal metastases. Therefore, minimally invasive spine treatment (MIST) (17), a concept describing the minimally invasive management of spinal diseases and including conservative treatment, radiotherapy, and less invasive conventional surgery, has been proposed.

In Section 4, we describe the conventional surgeries and the MIST including high-precision radiotherapy. Knowledge of cancer locomo, multidisciplinary approaches, and MIST can help spinal surgeons to overcome clinical inertia in cancer treatment (**Table 1**). Moreover, these findings herald a new era in the management of spinal metastases for spine surgeons.

**Table 1:** Three data sets of spinal surgeons overcoming clinical inertia in spinal metastases.

	Cancer locomo	Multidisciplinary approaches	MIST
Definition	A condition in which motor function is impaired due to cancer-related motor impairment: (1) directly affected by cancer (2) related to cancer treatment (3) coexisting with cancer	Multidisciplinary cancer board meetings for SRE prevention. Recommended preemptive treatment; prophylactic surgery, radiotherapy, administration of bone-modifying drugs, rehabilitation.	A comprehensive term for minimally invasive spine treatments, including radiation therapy, balloon kyphoplasty, and minimally invasive spine surgery.
Clinical inertia	Lack of engagement of orthopedic (spine) surgeons in cancer treatment including spinal metastasis	Multidisciplinary approaches to spinal metastases remain uncommon, even in cancer treatment-based hospitals	The disinterest of spine surgeons associated with cancer treatment and lack of updated knowledge regarding the latest treatments
Recommendations	Management of spinal metastases taking into account the “cancer locomo” perspective not only optimizes the quality of life of the patient, but also improves overall prognosis	Preemptive treatment with a multidisciplinary approach is essential to improve the environment nationwide, as it improves not only the quality of life but also the life expectancy of “cancer locomo” patients.	MIST expands the indications for treatment and improves the prognosis of patients who would otherwise be ineligible for conventional invasive surgery

skeletal-related events (SRE), minimally invasive spinal treatment (MIST).

## 2 Locomotive syndrome in cancer (“cancer locomo”) including spinal metastasis

Since 2007, the Japanese Orthopedic Association has advocated “locomotive syndrome,” a condition in which motor functions are weakened caused by musculoskeletal disorders, and has promoted the “locomotive syndrome prevention” movement (18). The locomotive syndrome is characterized by impaired mobility due to muscle weakness and musculoskeletal disorders, whereas patients frequently requires nursing care (18). The need to manage musculoskeletal dysfunction in patients with cancer is increasing owing to an increase in the population of patients with cancer following advances in cancer treatment. Therefore, orthopedic surgeons are increasingly required to actively participate in the treatment of cancer, a term coined by the Japanese Orthopedic Association in 2018, building on the concept of locomotive syndrome (1).

Locomotive syndrome in patients with cancer defined as a reduced motor ability due to cancer-related motor dysfunction and was categorized by three categories:

Type 1: Cancer-Induced Locomotive Dysfunction
  This category encompasses complications arising directly from the cancer such as bone metastasis, bone and soft tissue sarcoma, and cachexia, all of which affect the musculoskeletal system.
Type 2: Treatment-Related Locomotive Dysfunction
  Locomotive dysfunctions as a consequence of cancer therapy. These include muscle weakness due to prolonged sedentary care, secondary osteoporosis, peripheral neuropathy, lymphedema, and joint contractures due to oncological treatment.
Type 3: Concurrent Locomotive Dysfunction in Cancer Patients

This category includes locomotive issues that coexist with cancer, including osteoporosis, lumbar spinal canal stenosis, and osteoarthritis. These issues are sometimes inadvertently overlooked or undervalued in the orthopedic domain because of their primary focus on cancer.

Type 3, ostensibly tangential to cancer, is clinically important as such issues can be overlooked or underprioritized by orthopedic specialists with different oncological concerns (1). To address the overlooked or under-prioritized type 3 cancer-related motor impairments, the “Cancer Locomo” campaign advocates for a more inclusive role of the orthopedic surgeons in the cancer care. This campaign emphasizes the need for comprehensive locomotor management in patients with cancer, and ultimately aims to preserve patient autonomy and QOL, even for incurable or terminal cases.

Spine surgeons frequently address degenerative diseases, which often result in disengagements with oncological care and limited involvement in cancer treatment. This detachment may lead some spine surgeons to eschew involvement in cases involving patients with cancer, perceiving themselves as lacking expertise. Consequently, patients with cancer and comorbid locomotive dysfunction may miss the chance for adequate care because of their cancer diagnoses.

The principle of cancer rehabilitation institutionalized in Japan’s health insurance system in 2010 is dedicated to bolstering resilience and enhancing or sustaining functional capacity in patients with cancer (19). Conversely, campaigns addressing locomotive syndrome in cancer patients highlights the crucial role of maintaining and improving mobility through specialized management strategies. For example, while cancer rehabilitation might concentrate on adaptive training (such as wheelchair transfers) following pathological fractures, the Cancer Locomo approach advocates for aggressive surgical procedures such as internal fixation, total joint replacement, and spinal instrumentation to reinstate ambulatory capabilities. Collaborative synergy between cancer rehabilitation and Cancer Locomo can significantly enhance the ADL and QOL of patients with cancer. In general, limitations in daily activities caused by motor impairment also impact PS. Although poor PS in cancer treatment is generally a contraindication to chemotherapy and may lead to the discontinuation of cancer treatment, orthopedic surgeons have the possibility to correct the apparently poor PS due to motor impairment and expand the indications for cancer management further. In addition, pre-operative exercise may play a critical role in ensuring that patients achieve curative tumor resection; hence, leading to improved surgical outcomes and enhanced long-term survival (20). Therefore, managing spinal metastases from the Cancer Locomo perspective in cancer patients is critical not only for optimizing their QOL but also for improving their overall prognosis.

Insufficient recognition of the “Cancer Locomo” may result in clinical inertia among spinal surgeons when managing spinal metastases. A deeper understanding and more assertive intervention in Cancer Locomo can enhance not only the QOL of these patients but also their overall prognosis.

## 3 Multidisciplinary approaches for spinal metastasis

Recently, the management for spinal metastases has shifted owing to multimodal cancer therapy advancements, including advancements in chemotherapy, molecular targeted therapy, immune checkpoint inhibitors, BMA such as zoledronic acid and denosumab, and intensity-modulated radiotherapy, which can prevent spinal cord exposure (3). Hence, the aims of treatment for spinal metastases is changing from conventional palliative care to present QOL- or ADL-preserving care (1, 21).

SREs, such as bone metastases-related skeletal complications, defined as pathological fractures, spinal cord compression, necessity for radiation to the bone (due to painful or impending fracture), or surgery to the bone and are now recognized as factors that may affect the QOL and ADL of patients (22). The occurrence of SRE has been suggested to lead to worse prognosis, ADL disturbances or QOL deterioration (16, 23, 24). Thus, a primary objective in treating spinal metastases is to avoid pain, mechanical instability, and neurological deficits. As the SRE occurrence rate in patients with spinal metastasis was reported to be approximately 20% (23, 24), SRE prevention is gaining attention (25–28). Among SREs, symptomatic metastatic epidural spinal cord compression (MESCC) may result in nearly 20% of patients with spinal metastasis (23, 24). Watanabe et al. (29) and Helweg-Larsen et al. (30) described that the intensity of preoperative paralysis strongly influenced final walking ability. That is, once MESCC-induced severe spinal palsy occurs, the post-operative recovery and neurological prognosis do not improve. Therefore, screening and early diagnosis of spinal metastases and appropriate timing of surgery or radiation therapy are important for patients with cancer. Preventing subsequent complications such as MESCC and preserving ADL, including neurological function, may improve overall survival (31).

For cancer patients with bone metastasis, the significance of a multidisciplinary approach in the diagnosis, treatment, preservation, and ADL improvement has been recommended (1, 32, 33). In general, oncologists are constrained by their expertise in initial diagnosis and treatment of spinal metastases. However, multidisciplinary cancer board meetings on bone metastases are usually attended by primary cancer physicians, medical oncologists, orthopedic surgeons, spine surgeons, diagnostic radiologists, radiation oncologists, physiatrists, palliative care physicians, psycho-oncologists, physical therapists, occupational therapists, pharmacists, nurses, and medical social workers (32).

Preemptive treatment using a multidisciplinary approach includes prophylactic surgery, radiotherapy, and the administration of appropriate bone-modifying drugs to patients in need. Multidisciplinary approaches are reported to have a key role in patients with spinal metastases as they might lead to earlier recognition of a neurological deficit, initiation of radiological investigations, and treatment (33) Preservation of walking ability can be achieved by using radiotherapy for spinal cord metastases before walking function declines (19, 34), fractures and paralysis can be prevented through early detection of bone metastases via imaging surveillance even in the absence of subjective symptoms (20, 35), and paralysis as well as surgery can be prevented by timely interventions of a liaison team before SREs occur (21, 26). Maintaining the ability to walk at the terminal stage (30 days prior to death) (21), reduced costs and hospitalization (10–16), and improvement of the rate and severity of neurological impairment subsequent to local treatment (26). These ultimately improvement the overall survival (26, 32, 36).

Furthermore, a multidisciplinary approach is expected to contribute to the understanding and awareness of cancer locomo. In Japan, although the benefits of preemptive medicine with a multidisciplinary approach for patients with spinal metastases have become apparent, such multidisciplinary approaches are not common even in cancer hospitals (34). This might be explained by clinical inertia from spinal surgeons that are involved in the management of spinal metastases. Preemptive treatment using a multidisciplinary approach improves not only the QOL of patients with cancer locomo but also their life expectancy. Therefore, nationwide environmental improvements are required.

## 4 Advances in treatments for spinal metastasis

Surgical interventions for metastatic spinal tumors include radical (curative), pre-emptive prophylactic, and palliative interventions, with treatment options based on various decision-making systems. Surgical indications for metastatic spinal tumors include refractory pain, progressive palsy, and/or the development of bladder or bowel disturbances due to mechanical instability of the spinal structures and/or spinal cord/cauda equina/nerve root compression due to metastatic tumor invasion. Surgical interventions should be performed under tolerable general conditions rather than considering only life expectancy, which should longer than 3–6 months.

For patients with spinal metastases and a favorable PS, the absence of metastases to major organs, and tumors confined to the vertebral body, radical surgery including total en-bloc spondylectomy (TES), for complete removal of the tumor is preferred (35, 37). Based on tumor localization, less invasive radical surgeries including sagittal en-bloc spondylectomy and contralateral osteotomy of the pedicle and posterolateral elements for en-bloc resection (COPPER) have also been reported (38–40).

However, the number of patients who can be treated with radical surgery is limited due to the invasive and technical demands of the intervention. Recently, long-term local control has become possible by combining palliative surgery with radiotherapy and drug therapy (anticancer drugs and bone-modifying agents). Preemptive prophylactic surgeries and palliative interventions such as posterior fixation and decompression (41), separation surgery (42) and balloon kyphoplasty (BKP), including percutaneous vertebroplasty (PVP) (43), have been reported. Minimally invasive spine stabilization surgery (MISt) using percutaneous pedicle screw (PPS) fixation, which has been conducted commonly in Japan since 2005, applies to cancer patients in poor general condition (27). MISt is a minimally invasive fixation method that involves temporal stabilization of the spine using a PPS, which alleviates pain, promotes early ADL improvement, and prevents pathological fractures.

Approaches that render the management of spinal metastases less invasive, including radiotherapy, BKP, and MISt, are collectively referred to as MIST (17). MIST can provide an improvement in prognosis for patients ineligible for conventional invasive surgery (17, 27). MIST have expanded the indications for surgery and become essential for patients with cancer. If spinal surgeons lack the knowledge and interest in MIST approaches for spinal metastases, this could be considered clinical inertia. This section outlines decision-making systems and the management of metastatic spinal tumors.

### 4.1 Decision-making systems for managing spinal metastases

Patients with spinal metastases might experience various side effects, such as severity of pain, paralysis, aggressiveness of the cancer of origin, other life-threatening metastases, or general conditions that need to be considered for each patient.

Therefore, decision-making systems for managing spinal metastases can help patients and surgeons determine whether or how surgical interventions should be performed. Decision-making systems for managing spinal metastases are mainly divided into classification- and principle-based (44, 45).

#### 4.1.1 Classification-based decision-making systems

Traditionally, classification-based decision-making systems, such as Tomita (46), Tokuhashi (47), Bauer (48), and Katagiri (49) scoring systems, have been established and used to estimate survivorship in patients with spinal metastases.

However, recent cancer therapeutic developments, such as novel chemotherapies, hormonal therapies, molecular targeted therapies, and immune therapies, have rendered these scoring systems inaccurate (50–53). Thus, the Bauer scoring system was recently modified into the New England Spinal Metastasis Score (NESMS) (54). NESMS has been validated retrospectively (55, 56) and prospectively (57, 58). Recently, one study (59) reported that the NESMS had a better predictive estimation of the survivorship in patients with metastatic spinal tumors than the Tomita, Tokuhashi, and Spinal Instability Neoplastic Score (SINS) (60).

Within the clinical setting, spinal instability is commonly assessed using the SINS of spinal metastases (60). The SINS consists of a score that combines six variables: location of the lesion, pain, bone lesion(lytic/blastic), spinal alignment, degree of vertebral destruction and posterolateral involvement. In addition, the SINS provides an independent factor for the surgical indication of metastatic spinal tumors and is frequently used for decision-making. Of 18 points, a score of ≤6 indicates stability, whereas a scores of 7–12 or ≥13 indicate impending instability and instability, respectively (28, 60). Kakutani et al. recommended that patients with SINS of 10 or higher are at high risk of developing symptomatic spinal metastasis, suggesting consideration of interventions to prevent symptomatic spinal metastasis if a long-term prognosis could be expected (61).

Patients with higher SINS (≥10 points) tend to undergo stabilization surgery more frequently compared to patients with a SINS <9 (62).

Furthermore, another study found that patients with a SINS ≥ 10 points showed an increased risk of SREs despite the administration of denosumab (63).

#### 4.1.2 Principle-based decision-making systems

The NOMS is a principle-based decision-making systems (11). The NOMS framework comprised neurological (N), oncological (O), mechanical (M), and systemic (S) components. Among these, the neurological factor was assessed with the Bilsky grade (64). In contrast, the mechanical factor was assessed using the SINS (60).

In addition, Paton et al. modified the NOMS framework to the LMNOP system by adding two components: the location, levels, and number of metastases (L) as well as the responsiveness to previous treatment (P) (65).

#### 4.1.3 Contraindications

Contraindications for surgical intervention for spinal metastases should be considered in case of dissemination of carcinomatosis of the bone marrow (DCBM), excess multiple bone metastases, and poor performance or general condition. If these conditions are met, surgical procedures can be performed on patients with visible symptoms of metastatic spinal cord compression or mechanical instability.

In summary, with recent advances in treatment, no clear indications for surgery affecting life expectancy have been identified. Biological therapies, including molecular targeted therapies and immunotherapies, are considered new game-changers in modern cancer treatment. The application of decision-making systems to these evolving therapies will lead to the selection of more specific and tailored surgeries (radical and *in situ*).

### 4.2 Radical and palliative treatment for spinal metastases

#### 4.2.1 Radical treatment for spinal metastases

Patients with spinal metastases may be targeted for radical surgery if long-term survival is expected. TES, first reported by Roy-Camille et al. (66) and widely disseminated by Tomita et al. (66), is a common surgical technique used for the en-bloc resection of spinal tumors (67). Although TES is common practice worldwide, it has the potential for high-grade invasiveness. Consequently, according to the localization of the tumor, less invasive radical surgeries such as a sagittal spondylectomy or the COPPER method have been the procedures of choice.

**Figure 1:**
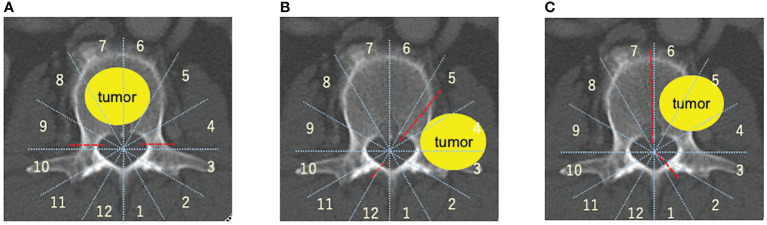
Spinal metastasis radical surgery based on the Weinstein-Boriani-Biagini (WBB) classification (67, 68). **(A)** TES, TES is mainly used for resection of entire vertebral body. **(B)** COPPER/modified COPPER, COPPER/modified COPPER can resect the spinal metastasis within zone 1-5, 7-12 based on the WBB classification. **(C)** Sagittal en-bloc resection, Spinal lesion located within the vertebral body, the pedicle, the transversal process, or paravertebral lesions within zone 2-5, 7-11 based on the WBB classification is good adaptation for sagittal resection. total en bloc spondylectomy (TES), contralateral osteotomy of the pedicle and posterolateral elements for en bloc resection (COPPER).

The Weinstein-Boriani-Biagini (WBB) classification is utilized to spinal tumor localization (67, 68) TES is performed in cases with tumors mainly located in the vertebral body within zones 5–9(**Figure 1A**)., the sagittal spondylectomy is used within the vertebral, pedicle, transversal process, or paravertebral lesion within zones 2–5, 7–11(**Figure 1B**)., whereas the COPPER method is used within zones 1–5 and 8–12 (**Figure 1C**).

##### 4.2.1.1 Total en-bloc spondylectomy

TES for spinal metastasis involves a surgical procedure aimed at removing the entire affected vertebra and adjacent discs as a single unit (35, 68). This technique is typically used in cases of aggressive spinal tumors or metastases involving the vertebral column. TES aims to achieve complete tumor removal, leading to improved local control. This is usually considered in cases of spinal metastasis where the tumor has extensively invaded the vertebral body (69). Some studies have found that TES may contribute to better survival rates in select cases, particularly when dealing with solitary or oligometastatic lesions (70–78).

TES is usually indicated in selected spinal metastasis patients with the following criteria: (1) single solitary, usually not more than three contiguous levels, spinal metastasis; (2) more than 1–2 years of life expectancy; (3) selected tumor histology (e.g., renal cell carcinoma, thyroid, breast, prostate, mucin-producing carcinoma, and some types of lung cancer); and (4) good pre-operative functional status (preferably an ECOG score 0-1) (35, 37, 68, 69, 79–82).

TES can remove the entire tumor in one piece, thereby reducing the risk of remaining cancerous cells. Thus, it provides a higher local disease control than that of other surgical techniques (83). However, due to the complexity of the procedure, TES requires sophisticated surgical techniques and expertise in spinal surgery to achieve favorable outcomes. TES has potential complications including neurological deficits, infections, and instrumentation-related issues (79, 84–88). The most common complications are massive intra-operative blood loss and a relatively longer operative time (84, 89, 90). To reduce the risk of bleeding intraoperatively, presurgical embolization is recommended (84, 89). However, even without embolization, TES can be safely performed with great care during meticulous hemostasis and careful surgical dissection (91). Thus, the overall complication rate can vary, and careful patient selection is crucial to minimize risks.

Furthermore, TES may result in effective pain reduction and improve QOL in patients with spinal metastases (77, 92). When performed successfully, TES can help to maintain or even restore neurological function. Patients often experience a reduction in pain and an improvement in their ability to perform daily activities post-operatively.

Advances in imaging, surgical instrumentation, and adjuvant therapies have continued to influence the evolution of TES techniques, potentially improving outcomes and reducing complications.

In summary, TES for spinal metastases represent a complex surgical procedure with the potential for significant benefits in selected cases. However, careful patient selection and thorough understanding of the associated risks and challenges are essential for successful outcomes. Multidisciplinary collaboration and ongoing research are crucial for refining the techniques and improving the overall management of spinal metastases.

##### 4.2.1.2 Representative case TES

**Figure 2:**
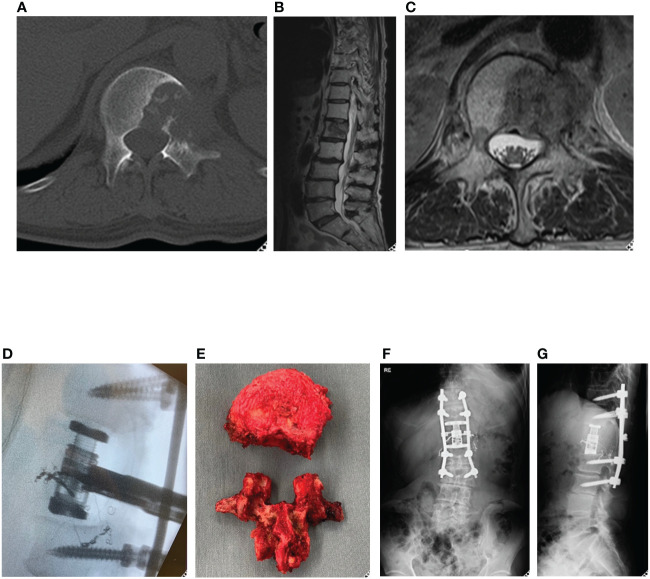
Representative case (total en bloc spondylectomy). 70-year-old man, primary left lung cancer, L1 Spinal metastasis. Axial CT **(A)** and T2-weighted MRI sagittal **(B)** and axial **(C)** revealed osteolytic lung cancer metastasis in the left pedicle of the L1 vertebral body. A single posterior approach total spondylectomy was performed **(D)**. For reconstruction of the anterior column, an expandable cage was inserted posteriorly, followed by posterior fixation **(E-G)**. total en bloc spondylectomy (TES).

A 70-year-old male was diagnosed with primary left lung cancer during a health examination. The patient underwent post-operative chemotherapy. One year following lung surgery, he developed lower back pain. CT and MRI scans suggested a metastatic lesion (**Figures 2A–C**), which lead to a referral to the spine surgery department.

Positron emission tomography-CT showed that the lesion in the L1 vertebral body was a solitary metastasis, and TES was planned as a curative surgery. TES was performed after arterial embolization (**Figures 2D–G**). Chemotherapy was continued, and subsequently the patient showed no evidence of the disease in the next three years.

##### 4.2.1.3 Sagittal en-bloc resection

Sagittal en-bloc resection is used for patients with spinal metastases located within the vertebral body, the pedicle, the transversal process, or paravertebral lesions within zone 2-5, 7-11 based on the WBB classification. First, the spinal surgeons remove the laminae and pedicles that did not invade the tumor. Next, unilateral costotransversectomy, ligation of never-roots (if needed), and release between the dura and the tumor are performed. The caudal and cephalad discs are transected with a bone scalpel. Osteotomy of the normal side of the spine follows the sagittal plane of the vertebrae with an ultrasonic scalpel. The tumor is completely resected from the spine. Dang et al. examined the feasibility, safety, and outcome of this surgical procedure for paravertebral tumors and supported its use (38).

##### 4.2.1.4 Contralateral osteotomy of the pedicle and posterolateral elements for en-bloc resection

Vasudeva et al. introduced COPPER as a novel surgical procedure (39). For this surgical procedure, laminectomies are performed above and below the tumor to reveal the dura, with a contralateral hemilaminectomy connecting them. The ipsilateral pars interarticularis and facet joints superior and inferior to the tumor are removed. The pedicles and nerve roots on the same side as the tumor are exposed, and an osteotomy is performed using a contralateral approach. This can produce tumor-free margins in all cases and was concluded that the COPPER approach was safely and effectively used for the en-bloc resection of tumors located in the posterior elements.

Toda et al. reported three cases of spinal and paravertebral tumors with anterior and extravertebral extension that were resected by the modified COPPER approach (40). In their report, due to the resection of soft tissue and multilevel osteotomy, the estimated operative blood loss and operation times were much greater than those of Vasudeva et al. However, Toda et al. achieved complete negative margins in all cases. In conclusion, the COPPER and modified COPPER approaches are feasible and appropriate for the en-bloc resection of spinal metastases.

#### 4.2.2 Preemptive prophylactic surgeries and palliative interventions for spinal metastasis

Preemptive prophylactic surgery and palliative interventions for spinal metastases have been developed using BKP and MISt, usually in combination with radiation therapy.

##### 4.2.2.1 BKP

BKP refers to a minimally invasive treatment designed to stabilize vertebral compression fractures and consists of polymethylmethacrylate (PMMA) cement injections into the vertebral body. A systematic review has demonstrated that BKP can reduce pain and improve physiological and functional outcomes in patients with vertebral compression fractures caused by metastatic spinal tumors and multiple myeloma (43). The underlying mechanism through which BKP produces pain reduction is attributed to the stabilization of the vertebral body and recovery of vertebral height. Traditionally, pain relief has been attributed to an exothermic reaction during the curing of PMMA cement (93, 94). Consequently, the injected PMMA is thought to exert a cytotoxic effect on surrounding cells, contributing to post-BKP pain relief (43, 95, 96). However, Toda et al. offered new insights on this matter. They analyzed histopathological samples from vertebral bodies retrieved following TES in patients who underwent BKP (97). Their results demonstrated the proliferation of spindle-shaped cell tumors and the presence of viable tumor cells with atypical mitotic figures. Moreover, no signs of bone or nerve necrosis adjacent to the PMMA cement were observed (97, 98). However, the effect of the heat generated during the polymerization of PMMA cement on tumor cells remains unclear.

The major complications observed in BKP include cement leakage, occurring in approximately 6% of cases, and new vertebral fractures adjacent to the vertebral body where PMMA cement was injected, with an incidence rate of 7.9–12.4% (99). In conclusion, BKP provides a minimally invasive treatment that reduces pain and improves the QOL of patients with malignant vertebral compression fractures.

##### 4.2.2.2 Palliative surgery: conventional vs MISt

Conventional spine surgeries for spinal metastasis are highly invasive procedures that requires the selection of patients according to their prognosis and surgical complications. They are associated with long hospitalization and its clinical effectiveness is debated. MISS techniques have been adopted for spinal metastases, including kyphoplasty/vertebroplasty, percutaneous fixation, tubular retractors, mini-open procedures, and thoracoscopy/endoscopy (100).

##### 4.2.2.3 Conventional procedures

The surgery for spinal metastasis usually involves posterior decompression and stabilization with a median incision through the fixation level. Direct decompression with or without instrumentation and radiation have been shown to be superior to radiation alone (95). Although not curative, surgery can alleviate pain and related complications and improve the patient’s quality of life (96, 101). The beneficial effects of surgery for spinal metastases were observed regardless of age. Based on an analysis of 914 patients who received debulking (tumor resection of <50% of the tumor volume) and instrumentation surgery for symptomatic spinal metastases, these surgeries are recommended for patients with a longer estimated survival to benefit from reduced pain, better or sustained neurological function, and better QOL (102).

##### 4.2.2.4 MISt procedures

Currently, PPS is utilized for long-segment MIS spinal fixation from the thoracic spine to the pelvis in pathological conditions, including spinal metastasis (15, 25). The benefits of MISt include a small skin incision and reduced tissue invasiveness. Smaller incisions result in bleeding during the perioperative period and less need for blood transfusions (103, 104). Furthermore, an advantage of the small incision is that early post-operative radiotherapy and adjuvant therapy can be initiated more rapidly (105, 106). The lack of updating and committing to MISt represent clinical inertia in spinal metastasis management. Therefore, we discuss the recent MIST procedures, including MISt and radiotherapy.

##### 4.2.2.5 Comparison between conventional procedures and MISt

Comparative studies of minimally invasive and open surgery have shown that minimally invasive surgeries provide similar or superior outcomes with decreased blood loss, surgical morbidity, and complications in patients with spinal metastases (107–110).

Hikata et al. reported that patients with MISt showed significantly less blood loss, less post-operative drainage, lower rates of blood transfusion and complications, shorter bed rest periods, and lower complication rates than those with conventional procedures (111).

Comparisons of conventional posterior decompression and stabilization using MISt have demonstrated that MISt has a smaller surgical wound, shorter operative time, and less intraoperative blood loss compared to the conventional method (30, 111–113).

According to a recent systematic review of 26 studies, MISt has the potential to reduce surgical site infection, hospitalization, and bleeding in patients with spinal metastases without compromising instrument accuracy or overall patient outcomes (105). This indicates that MISt could be utilized in more cases, including the elderly patients with a prognosis of 6 months or less That is, MISt provides opportunities for patients who are not eligible for conventional surgery because of the method-associated invasiveness.

A key drawback of MISt is the challenge associated with bone transplantation. Atanasiu et al. (106) found that bone transplantation is necessary for patients with a life expectancy of 2 years or more. Nakanisi et al. (27) found that no patients needed further surgery with temporary fixation using MISt and concluded that it was suitable until unstable lesional vertebral bodies were remodeled and stabilized.

Morgen et al. (107) performed a survival analysis and compared patients receiving MISt and OS procedures and observed no significant differences between them, even though blood loss was significantly reduced following MISt. As the quality of evidence in the present literature is deemed to be low, no clear conclusion concerning the advantages or disadvantages of minimally invasive surgery over open surgery can be derived, and no strong recommendations have been made at this time (100). Therefore, to prove the benefits of the MISt procedure for spinal metastases, further clarifications are needed.

#### 4.2.3 Radiotherapy for spinal metastasis

Spinal radiotherapy was first used in the 1950s and several articles have shown that no benefit of combining laminectomy and radiation therapy compared to radiation therapy alone (108, 109). Prior to the advanced radiotherapy techniques, conventional external beam radiation (cEBRT) was primarily utilized to enhance the local control during surgery (110). During the 1980s, direct decompression surgery with spinal reconstruction was invented and the importance of surgery was reconsidered (95, 114). Several research has demonstrated better functional outcomes and pain relief following direct compression surgery combined or without postoperative radiotherapy than with radiotherapy alone (114, 115). In the randomized, multicenter, open-label study reported by Patchell et al. in 2005, patients undergoing direct decompression surgery combined with radiotherapy showed significantly higher post-treatment ability of walking (84% vs 57%, p = 0.001) and a prolonged duration of ambulatory period (median 122 days vs 13 days, p = 0.003) than those treated with radiotherapy alone (95).

Subsequently, new radiation therapy techniques such as stereotactic radiosurgery (SRS) and stereotactic body radiotherapy (SBRT) have been developed (116). SRS and SBRT are complex techniques in which radiotherapy is administered at high doses per fraction in a small number of fractions, usually 1–5 (116). These advances have also made it possible to create a steep dose “falloff” gradient of approximately 10% per millimeter around the target. Thus, maximum dose can be achieved at the target and dose to surrounding vital structures and healthy tissue can be minimized. In a palliative setting for control of symptoms in patients with painfully spinal metastases, SBRT was demonstrated to be linked to a superior complete response rate to pain in comparison to that of conventional radiotherapy (117).

##### 4.2.3.1 Separation surgery combined with radiotherapy

Advancements in radiation techniques have made separation surgeries possible. Separation surgeries are utilized as a surgical technique to separate the anterior sulcus in the spinal canal and posterior edge of the vertebral body (118). “Separation surgery” represents a surgical procedure in which tumor resection remains restricted to decompression of the spinal cord, creating a gap between the spinal cord and the tumor, making it a safe target for SRS (119).

Separation surgery combined with SRS can improve not only functional outcomes and pain relief, but also oncological outcomes (120). Laufer et al. performed separation surgeries combined with SRS under epidural compression in 186 patients with spinal metastases (121). They showed that the low-fractionated high-dose radiotherapy group (24–30 Gy/3fr) exhibited the lowest progression rate of 4.1% at one year compared to rates of 9.0% in the single high-dose (24 Gy/1 fraction) and 22.6% in the low-fractionated low-dose (18–36 Gy/5–6fr) groups. Xiaozhou et al. retrospectively investigated the clinical data of 52 patients with spinal metastases and found that postoperative SBRT combined with segmentectomy significantly improved survival up to 38 months compared with 21 months in patients who underwent surgery alone (122). A recent meta-analysis by Kang et al. (123) found that the a pooled local progression rate of 10.2% after 1 year of hybrid treatment, with factors such as low doses per fraction, previous radiotherapy, and colorectal cancers significantly associated with local tumor progression (123).

Gong et al., in their study of patients undergoing surgery and hypo-fractionated SBRT, discovered that patients with a post-operative epidural tumor-to-spinal cord separation of <3 mm had poorer local control compared to those with a separation of ≥3 mm. Therefore, maintaining a minimal distance of >3 mm can lead to reliable local control of the tumor.

The occurrence of pathological fractures after radiotherapy is of a concern. Fracture rates were reported in 10.4–39% of patients with spinal metastases who underwent SRS (124, 125). Among patients with primary spinal/paraspinal sarcoma, vertebral compression fractures occurred in 23% of those who received carbon-ion radiotherapy (CIRT) (126) and in 27.3% of those who underwent separation surgery combined with CRIT (127).

## 5 Conclusion

The ability to walk is important for extending the duration of life with cancer and for enabling cancer patients to live their own independent lives, continue to work, and continue cancer treatment until the end of their lives. Spinal metastases have a significantly impact on walking ability and spinal surgeons must be actively involved in the therapeutic process. However, orthopedic and spinal surgeons in Japan tend to avoid involvement with patients with cancer. Spine surgeons can overcome clinical inertia in cancer treatment by improving their knowledge of cancer locomotion, non-surgical treatment, multidisciplinary approaches such as cancer boards and clinical conferences, and various surgical procedures, including radical resection and MIST. Preserving the QOL and ALD of patients with cancer should be the mission of orthopedic and spinal surgeons.

## Author contributions

TM: Conceptualization, Data curation, Funding acquisition, Investigation, Methodology, Project administration, Writing – original draft, Writing – review & editing. YuT: Conceptualization, Data curation, Investigation, Visualization, Writing – original draft. MH: Conceptualization, Supervision, Writing – original draft, Writing – review & editing. PP: Conceptualization, Data curation, Formal analysis, Writing – original draft. KW: Conceptualization, Investigation, Validation, Writing – original draft, Writing – review & editing. KK: Data curation, Formal analysis, Investigation, Writing – original draft. MT: Supervision, Validation, Writing – review & editing. HH: Conceptualization, Methodology, Supervision, Validation, Writing – review & editing. YK: Conceptualization, Data curation, Formal analysis, Methodology, Supervision, Writing – original draft, Writing – review & editing. YaT: Conceptualization, Data curation, Investigation, Writing – original draft. SN: Project administration, Supervision, Validation, Writing – review & editing. KN: Formal analysis, Project administration, Supervision, Validation, Writing – review & editing. HK: Investigation, Supervision, Validation, Writing – review & editing. YM: Methodology, Project administration, Supervision, Validation, Writing – review & editing. KO: Data curation, Formal analysis, Methodology, Project administration, Supervision, Validation, Writing – review & editing. MM: Methodology, Project administration, Supervision, Validation, Writing – review & editing. TN: Data curation, Methodology, Supervision, Validation, Writing – review & editing.
